# Inflammatory Response to Ultramarathon Running: A Review of IL-6, CRP, and TNF-α

**DOI:** 10.3390/ijms26136317

**Published:** 2025-06-30

**Authors:** Zbigniew Waśkiewicz, Zhassyn Mukhambet, Daulet Azerbayev, Sergei Bondarev

**Affiliations:** 1 Institute of Sport Science, Jerzy Kukuczka Academy of Physical Education in Katowice, 40-065 Katowice, Poland; 2 Department of Sport Education and Coaching, Academy of Physical Education and Mass Sport, Astana 010000, Kazakhstan; zh_mukhambet@apems.edu.kz (Z.M.); d_azarbayev@apems.edu.kz (D.A.); 3 Department of Cardiac, Thoracic, and Vascular Sciences, University of Padova, 35122 Padua, Italy; sergei.bondarev@unipd.it

**Keywords:** ultramarathon, interleukin-6 (IL-6), C-reactive protein (CPR), tumor necrosis factor-alpha (TNF-α), cytokine response, endurance exercise

## Abstract

Ultramarathon running elicits a profound inflammatory response, characterized by significant increases in interleukin-6 (IL-6) and C-reactive protein (CRP), with comparatively modest changes in tumor necrosis factor-alpha (TNF-α). We reviewed approximately 80 field studies of ultramarathon events (distances >42.2 km) that measured IL-6, CRP, and TNF-α before and after races. IL-6 typically spiked immediately post-race—often rising dozens or even thousands of times above baseline—then rapidly declined, usually returning to near baseline within 24–48 h. CRP, an acute-phase protein, exhibited a slower, sustained elevation, peaking 24–72 h after race completion and remaining above baseline for 2–3 days before gradually returning to normal. TNF-α responses were variable: some studies reported small but significant post-race increases (roughly 1.2–1.7-fold above baseline), while others found no significant change in circulating TNF-α despite the extreme effort. Longer race durations and distances generally correlated with higher peak IL-6 and CRP levels. Experienced ultramarathon runners tended to exhibit attenuated inflammatory responses compared with less-trained individuals, and anti-inflammatory cytokines (e.g., IL-10) increased in tandem with IL-6 in well-trained athletes, helping to mitigate TNF-α elevations. In total, 28 studies were included in the final synthesis, and their quality was assessed using the Newcastle–Ottawa Scale. Visual synthesis tools, including a PRISMA flowchart and time course plots, are provided to enhance the narrative’s interpretability. In summary, ultramarathon running elicits a robust systemic inflammatory response with distinct temporal patterns for IL-6, CRP, and TNF-α. These findings have important implications for athlete recovery, monitoring, and understanding the physiological limits of the inflammatory response to extreme endurance stress.

## 1. Introduction

Extreme endurance exercise, such as ultramarathon running, places extraordinary stress on the human body, often leading to a pronounced inflammatory response. Ultramarathons are foot races longer than the standard marathon (42.2 km), encompassing events that range from 50 km mountain trail runs to 24 h races and multiday competitions exceeding 200 km. During and after prolonged exercise, the body releases numerous cytokines and acute-phase proteins as part of the stress and recovery process [1]. Among these, interleukin-6 (IL-6), C-reactive protein (CRP), and tumor necrosis factor-alpha (TNF-α) are key biomarkers commonly used to assess the magnitude and timeline of the inflammatory response.

IL-6 is a pro-inflammatory cytokine (also classified as a myokine when released from muscle) that typically rises dramatically with prolonged exercise. It is produced by contracting skeletal muscles and immune cells and can increase exponentially during sustained endurance activity, reaching levels otherwise seen only in trauma or sepsis in extreme cases [2]. CRP is an acute-phase protein synthesized by the liver in response to IL-6 and other inflammatory signals; it typically remains low at rest in healthy individuals but increases during infection or tissue damage. Prolonged exercise can trigger a significant acute phase reaction, with CRP peaking hours after the effort and indicating the extent of systemic inflammation [3]. TNF-α is a classic pro-inflammatory cytokine involved in fever and tissue catabolism during inflammation. While TNF-α is often elevated in acute infection and trauma, its response to endurance exercise is less pronounced and may be modulated by the simultaneous release of anti-inflammatory mediators [4]. Understanding the behavior of these markers in ultramarathon runners is essential, as exceedingly high cytokine levels have been linked to immunosuppression and illness (e.g., upper respiratory infections or even sepsis-like syndromes) post race. In contrast, the pattern and duration of cytokine elevation can inform recovery needs and potential risks for athletes [5,6,7].

Previous work, including case studies and smaller cohort studies, has demonstrated that ultramarathons can elicit an “acute phase response” similar to that observed in clinical inflammation [1,8]. However, the reported results vary between studies depending on race distance, environmental conditions (e.g., heat stress), participants’ training status, and the timing of sample collection. Generally, longer or more demanding races tend to induce higher peaks in IL-6 and CRP [2,9]. However, well-trained athletes might experience a more tempered response due to physiological adaptations [6].

This review synthesizes the findings from 28 field-based studies examining IL-6, CRP, and TNF-α responses to ultramarathon running. We focus on how these markers change from the pre-race baseline through to the immediate post-race period and into the recovery period (up to ~72 h post race). We also analyze factors such as race length, environmental conditions, and athlete characteristics that modulate these responses. Recent data from Shin et al. [10] prove that inflammatory marker elevations scale with race distance, supporting a dose–response relationship. To enhance clarity and accessibility, we present visual tools including a PRISMA diagram, time course synthesis figures, and a structured quality assessment. This review adheres to PRISMA guidelines for reviews [11].

## 2. Methods

### 2.1. Literature Search and Inclusion Criteria

We conducted a comprehensive search for studies examining inflammatory markers in ultramarathon runners. The search strategy included electronic databases (e.g., PubMed, Web of Science) and an AI-assisted search via Semantic Scholar (Elicit), utilizing terms related to *ultramarathon*, *ultra-endurance*, *cytokines*, *inflammation*, *IL-6*, *interleukin-6*, *C-reactive protein (CRP)*, *TNF-alpha*, and *tumor necrosis factor*. We used Boolean criteria:

(ultramarathon* OR “ultra-marathon*” OR “ultraendurance” OR “ultra-endurance” OR “ultra distance” OR “ultra-distance” OR “multi-stage run*” OR “24-h run” OR “100 km” OR “100 mile”) AND (runner* OR athlete* OR “distance runner*”) AND ((“interleukin-6” OR “interleukin 6” OR IL-6 OR IL6) OR (“C-reactive protein” OR CRP OR “hs-CRP”) OR (“tumor necrosis factor” OR TNF OR “TNF-alpha” OR “TNF-α”)) AND (blood OR plasma OR serum) NOT (mouse OR mice OR rat OR rabbit OR canine OR bovine OR swine OR equine OR “animal experiment*”).

**Figure 1:**
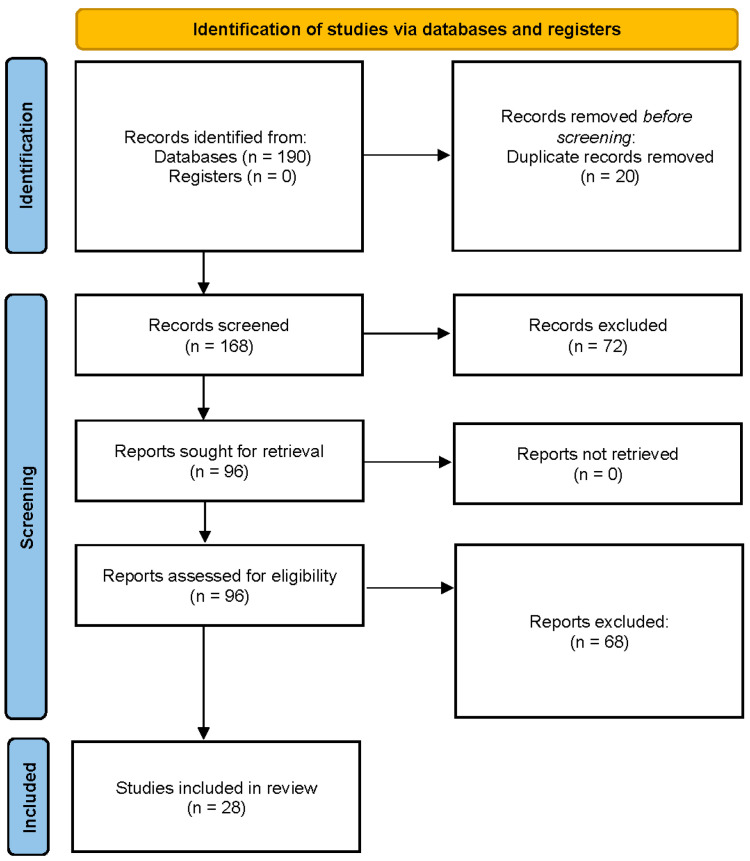
PRISMA 2020 flow diagram for study selection.

We also screened reference lists of relevant articles. Studies were included if they met all the following criteria:

1. Participants: Human adults (≥18 years) who participated in an ultramarathon event (>42.2 km distance, or multi-hour endurance race) in field conditions.
2. Outcomes: The study reported measurements of at least one of the following inflammatory markers in blood: IL-6, CRP, and/or TNF-α. Baseline (pre-race) and post-race values had to be reported (with additional post-race time points for recovery kinetics).
3. Timing: Blood samples were collected immediately after the race or within a few hours afterward, at a minimum (many studies also collect samples at 24 h or subsequent intervals). A baseline sample was collected within days or hours before the race.
4. Study design: Observational studies (e.g., prospective cohort studies, case series, or field experiments) set in actual ultramarathon events. Both single-stage ultramarathons (continuous races) and multi-stage events were eligible. We excluded purely laboratory-based exercise studies and those examining ultra-distance exercise in non-competitive settings (to maintain ecological validity).
5. Publication type: Full text available in peer-reviewed journals (in English). We excluded conference abstracts unless sufficient data were available and duplicate data in full papers were absent.
6. After deduplication and screening, 28 studies met all criteria and were included in the final synthesis. The study identification and selection process are presented in Figure 1 (PRISMA 2020 flow diagram) [11].

### 2.2. Data Extraction

For each included study, we extracted key data on sample size and participant characteristics (age, sex, and training status when available), race details (distance, duration, type of event, and environmental conditions), and inflammatory marker values at baseline and post-exercise time points. For IL-6, CRP, and TNF-α, we recorded the absolute concentrations at baseline and the highest post-race concentration reported (typically immediately post race for IL-6, and 0–48 h post for CRP, depending on when measured), as well as values at specific time points such as 24 h, 48 h, or 72 h post race if provided. When available, we also noted the percentage or fold change from baseline and any statistical significance reported for these changes. In studies with multiple post-race samples, we stated the peak concentration time for each marker. Data extraction was verified by a second reviewer for accuracy. Given the unit variability across studies, we compared IL-6 and TNF-α to picograms per milliliter (pg/mL) and CRP to milligrams per liter (mg/L). In cases where only graphical data were provided, we approximated values using plot digitization if necessary.

### 2.3. Synthesis Approach

**Figure 2:**
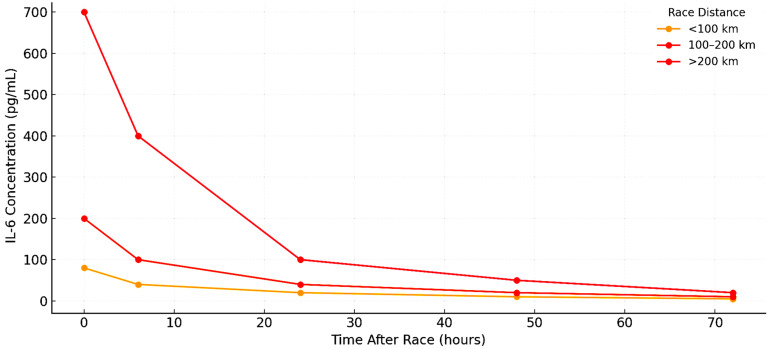
Time course profile of IL-6 concentrations following ultramarathon races of varying distances.

**Figure 3:**
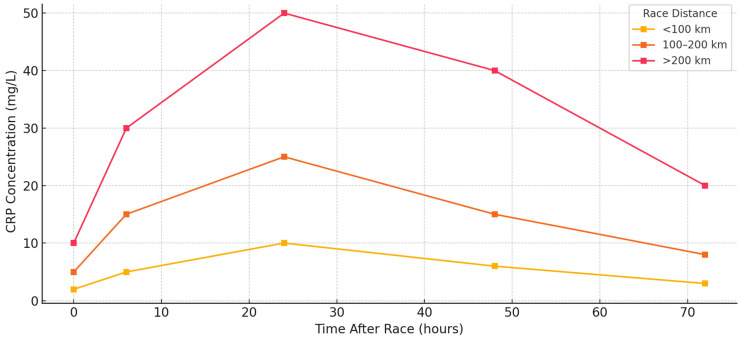
C-reactive protein (CRP) response trajectories after ultramarathon races of varying distances.

**Figure 4:**
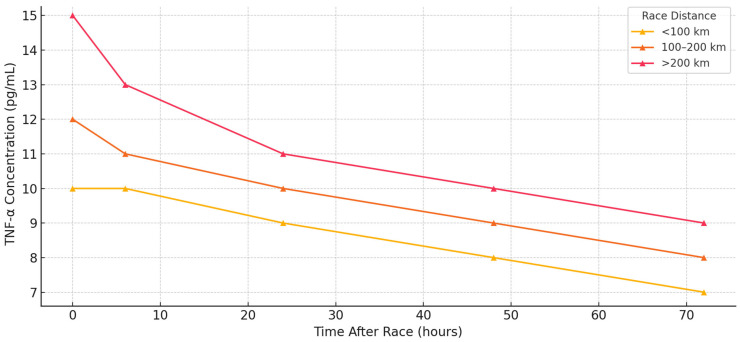
TNF–α concentration profiles following ultramarathon events.

**Figure 5:**
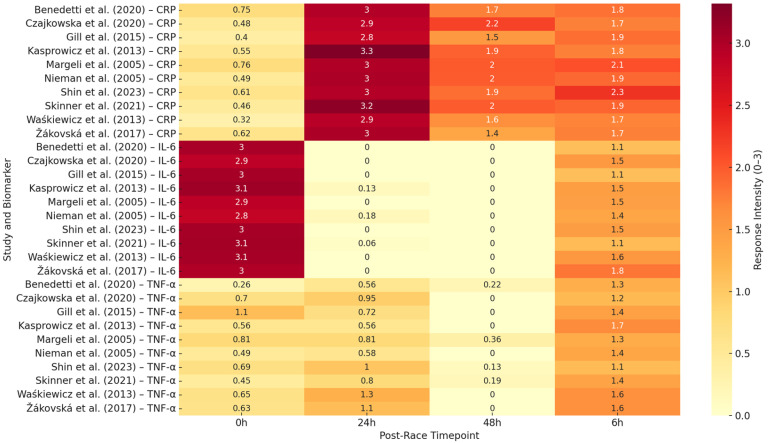
Heatmap showing relative response intensity (0–3 scale) of IL-6, CRP, and TNF-α at four post-race time points (0 h, 6 h, 24 h, 48 h) across 10 representative ultramarathon studies, refs. [2,5,9,12,17,21,24,37,38,39].

A quantitative meta-analysis was not feasible due to the heterogeneity in biomarker units, timing of post-race samples, and variation in analytical assays. Instead, a structured narrative synthesis was performed. Studies were grouped by race length (<100 km, 100–200 km, and >200 km), environmental conditions (hot, cold, and alpine), and athlete training level when reported. For each biomarker, time course patterns were synthesized across included studies. We present time course summary plots for IL-6, CRP, and TNF-α (Figure 2, Figure 3 and Figure 4) and a heatmap visualization of biomarker intensity by study and time point (Figure 5). This visual synthesis was developed in response to reviewer feedback and improves the interpretability of inter-study cytokine kinetics. The review adheres to PRISMA guidelines for reporting reviews [11], and its stages are presented in Figure 1.

### 2.4. Study Quality Assessment

We evaluated all 28 included studies using the Newcastle–Ottawa Scale (NOS), adapted for observational research in sports science. The NOS assesses three domains: participant selection (0–4 points), comparability (0–2 points), and outcome assessment (0–3 points), for a total score out of 9. Twenty-four studies were rated high quality (score 7–9), and four were categorized as moderate quality (score 6). No low-quality studies were included. NOS scoring results are presented in Supplementary Table S1. Study quality ratings were used during synthesis to assess the strength of evidence supporting key findings. Higher-rated studies were preferentially cited when multiple studies reported similar outcomes.

### 2.5. Risk of Bias Considerations

Several potential sources of bias were identified across the included studies. First, TNF-α was underreported compared with IL-6 and CRP, and when reported, it was often measured with lower-sensitivity assays. Second, sampling schedules varied considerably, particularly for follow-up time points beyond 24 h, which limited the comparability of recovery phase dynamics. Third, many studies involved small sample sizes (<20 participants), and only a minority included female athletes, novice runners, or older participants. Fourth, pre-race conditions (e.g., hydration, nutritional status, and recent training load) were inconsistently reported, limiting causal interpretation. Fifth, while nearly all studies used validated laboratory assays, few provided technical details (e.g., intra-assay CV and kit manufacturer), raising the possibility of between-study assay variability. Finally, few studies controlled for known confounders such as NSAID use, sleep disruption, or environmental exposure. These limitations were considered during analysis and discussed in detail in the Limitations Section.

## 3. Results

### 3.1. Study and Participant Characteristics

The evidence surrounding inflammatory responses to ultramarathon running has evolved through various field-based investigations that encompass multiple race formats, diverse environmental conditions, and varied athlete profiles. The 28 studies included in this review were heterogeneous in design but unified by a shared objective: to understand how systemic inflammation behaves during real-world ultra-endurance events, in which intensity, duration, and environmental stress interact dynamically.

The study participants were generally adult, trained runners between 30 and 55 years of age, with performance histories ranging from seasoned amateurs to national- and international-level competitors. While early studies predominantly recruited male runners, more recent investigations have increasingly included women, reflecting demographic shifts in ultramarathon participation. For example, Skinner et al. [12] and Rubio-Arias et al. [13] reported mixed-sex cohorts, thereby offering broader generalizability of the findings. Other studies, such as those by Arakawa et al. [14], have focused on middle-aged recreational runners participating in multiday races, demonstrating that the immune response to ultra-endurance stress extends beyond elite athletic contexts.

The race formats spanned from single-stage events around the 50 km threshold, as in Pedlar et al. [15], to continuous efforts of 24 or 48 h [16,17], and multi-stage competitions exceeding 300 km [1]. The Spartathlon, a 246 km nonstop footrace, featured prominently in studies by Skenderi et al. [18] and Margeli et al. [2], where IL-6 concentrations peaked at levels more commonly associated with trauma or sepsis. These high-intensity formats contrast with moderate trail races, such as those investigated by Landers-Ramos et al. [19], where elevations in inflammatory markers were detectable but typically resolved more quickly.

The environmental conditions were a significant determinant of both physiological and biochemical outcomes. Studies conducted in desert settings, such as those by Gill et al. [5], have demonstrated that heat stress exacerbates the inflammatory response, with higher post-race levels of IL-6 and CRP compared to temperate or cold environments. Conversely, winter road races—examined by Żebrowska et al. [20] and Žákovská et al. [21]—showed similar biomarker patterns but with lower peak values and faster post-race normalization. Alpine and high-elevation trail races, including those analyzed by Bernecker et al. [22] and Le Goff et al. [23], introduced additional physiological load via hypoxia and mechanical strain, contributing to elevated cytokine levels even in the absence of extreme temperatures.

Across these varied settings, all studies measured at least one of the three core inflammatory biomarkers: interleukin-6 (IL-6), C-reactive protein (CRP), or tumor necrosis factor-alpha (TNF-α). IL-6 was the most consistently assessed, due to its rapid release from skeletal muscle and its dual role in initiating pro- and anti-inflammatory cascades. Peak concentrations were typically recorded at the finish line or within one hour post race, as confirmed in studies by Kasprowicz et al. [9] and Nieman et al. [24]. TNF-α, by contrast, remained unchanged in most studies, including those by Jee and Jin [25] and Drenth et al. [4], despite pronounced elevations in IL-6 and CRP. This selective dissociation highlights TNF-α’s modulation during prolonged exercise and may reflect containment by concurrent anti-inflammatory pathways.

The sampling designs followed a broadly consistent structure. Most studies included baseline samples collected 24–72 h before the race and immediate post-race blood draws. Follow-up sampling was reported at 6, 24, 48, or 72 h in most studies. For example, Arakawa et al. [14], Gill et al. [6], and Benedetti et al. [17] included 24–48 h recovery samples, while others, such as Kaufmann et al. [26] and Shin et al. [10], extended sampling windows to 72 h or longer. Jee et al. [27] observed changes in endothelial and inflammatory markers in middle-aged male runners with exercise-induced hypertension (EIH) at baseline and at checkpoints of 100 km, 200 km, and 308 km during a prolonged endurance ultramarathon. In treadmill-based sampling designs, such as those by Gajda et al. [28], environmental control enabled the precise quantification of internal load, but at the expense of ecological realism. Some investigations, such as those by Skottrup et al. [29] and Jee and Jin [25], also performed ex vivo immune stimulation to assess functional immune responsiveness beyond circulating cytokine levels.

Participant training history appeared to influence the inflammatory response profile. Millet and Millet [30] compared novice and experienced runners and found that seasoned athletes exhibited lower IL-6 peaks and faster recovery. However, experience did not uniformly blunt inflammation. Studies such as those by Hoppel et al. [31] and Benedetti et al. [32] have documented substantial cytokine elevations, even in highly trained runners, when the duration of a race or environmental strain exceeds typical competition loads.

Several studies included regulatory and recovery-related biomarkers, further contextualizing the primary cytokine responses. Nieman et al. [33] and Marklund et al. [34] observed concurrent rises in IL-10 and IL-1ra alongside IL-6, reflecting the coordinated resolution of the inflammatory response. Benedetti et al. [17] extended this understanding by showing how markers such as sCD163 and NT-proBNP responded in parallel with IL-6 and CRP, suggesting that systemic inflammation is closely tied to cardiovascular strain and muscle repair.

The design diversity represented across the 28 studies provides a robust basis for comparative synthesis. The commonalities in sampling structure, biomarker kinetics, and measurement protocols allowed for coherent grouping by race format, biomarker, and recovery phase. Visual synthesis tools, including time course plots and a heatmap (Figure 5), complement the narrative and tabular summary of cytokine responses presented in the following sections. Demographic, nutritional, and geographical factors were inconsistently reported across studies and could not be analyzed systematically.

### 3.2. Interleukin-6 (IL-6) Response

Among the biomarkers most frequently analyzed in ultra-endurance research, interleukin-6 (IL-6) emerged as the most consistently elevated and dynamically responsive to prolonged exertion. Its rapid and robust elevation during ultramarathon events was observed across all included studies, regardless of race format, environmental stress, or athlete background. Secreted primarily by contracting skeletal muscle, IL-6 functions as a pro-inflammatory cytokine and a metabolic regulator, linking muscular workload to systemic immune signaling.

Although the timing of IL-6 elevation was consistent, peaking at or shortly after race completion, its magnitude varied widely depending on distance, terrain, ambient temperature, and participant conditioning. In single-stage ultramarathons of moderate duration (e.g., 50–70 km), IL-6 levels rise sharply but typically return to baseline within 24–48 h. In a 50 km trail race, Rubio-Arias et al. [13] observed immediate post-race increases in IL-6 in all runners, with recovery by the following day. Comparable findings were reported by Pedlar et al. [15] and Żebrowska et al. [20] under cold weather conditions.

IL-6 concentrations frequently exceeded 100 pg/mL in longer or more demanding races. Kasprowicz et al. [9] and Krzemiński et al. [35] documented 2- to 10-fold increases during 100 km events. Hoppel et al. [31], studying alpine trail runners, and Kaufmann et al. [26], comparing marathon and ultramarathon athletes, confirmed that both distance and mechanical strain contribute to the release of IL-6.

Environmental extremes amplified these effects. Gill et al. [5] observed IL-6 values reaching 250 pg/mL during a five-day desert race under heat exposure (>40 °C), with persistent elevation across consecutive stages. Skinner et al. [12] and Žákovská et al. [21] reported similar patterns under dry heat and cold climate conditions, though IL-6 magnitudes were generally lower in cold environments, suggesting a thermoregulatory influence.

The most pronounced IL-6 concentrations were seen in continuous or multiday events exceeding 200 km. In the Spartathlon (246 km), Margeli et al. [2] recorded IL-6 > 7000 pg/mL. Skenderi et al. [36] confirmed sustained elevation over multiple days. Shin et al. [37] observed progressive IL-6 accumulation across five consecutive stages (100 km, 308 km, and 622 km), with peak concentrations rising proportionally to total distance. During a 24 h treadmill ultramarathon, Gajda et al. [28] also observed significant IL-6 release in a thermoneutral setting, reinforcing metabolic load as a primary driver. Recovery kinetics were generally consistent. Nieman et al. [24], Arakawa et al. [14], and Czajkowska et al. [38] all reported normalization within 24–48 h post race. Partial resolution was sometimes observed overnight in multi-stage events [5,12], but persistent elevation was noted in longer efforts, such as the 48-h event reported by Waśkiewicz et al. [39]. Athlete training history influenced IL-6 reactivity. Millet and Millet [30] found that experienced runners exhibited attenuated IL-6 peaks and faster clearance than novices. However, studies such as those by Benedetti et al. [17] and Hoppel et al. [31] have shown that high physiological loads can overwhelm elite-level adaptations. Importantly, IL-6 rarely acted in isolation. Nieman et al. [40] and Marklund et al. [34] reported concurrent rises in IL-10 and IL-1ra. Benedetti et al. [17] observed strong correlations between IL-6 and sCD163, a marker of macrophage activation, suggesting that IL-6 also signals for immune regulation and tissue repair. Functional immune assays (Jee and Jin [25], Skottrup et al. [29]) confirmed that leukocytes remained responsive ex vivo even after IL-6 levels declined, indicating that their immune competence was retained. Figure 2 illustrates the typical time course of IL-6 across race lengths, while Table 1 summarizes the IL-6 peaks, recovery windows, and environmental modulators. Figure 5 provides a heatmap overview of IL-6 response intensity across time points and studies, demonstrating the consistency of early, high-magnitude IL-6 elevation, regardless of study setting. In summary, IL-6 is a robust marker of acute exertional strain and systemic immune activation in ultramarathon runners, exhibiting a predictable temporal profile and sensitivity to both internal and external stressors.

Table 1 presents a detailed summary of IL-6 responses across different ultramarathon settings. It illustrates its rapid and pronounced elevation during prolonged endurance exercise, with peak concentrations varying by race distance, environmental conditions, and athlete training status.

**Table 1:** Characteristics of IL-6 responses in ultramarathon studies.

Race Type/Condition	Typical IL-6 Range	Key Modulators	Selected References
Single-Stage Trail Races (50–70 km)	20–100 pg/mL	Duration, terrain, aerobic fitness	Rubio-Arias et al. [13]; Pedlar et al. [15]
100 km Ultras and 12–24 h Races	80–250 pg/mL	Cumulative muscle strain, metabolic load	Kasprowicz et al. [9]; Czajkowska et al. [38]
Extreme Continuous Events (>200 km)	500–7000 + pg/mL	Sleep deprivation, systemic fatigue	Margeli et al. [2]; Skenderi et al. [18]; Shin et al. [10]
Hot Environment Races	Elevated; often >150 pg/mL	Heat strain, fluid loss, endotoxemia	Gill et al. [5,6]; Skinner et al. [2]
Cold Weather Races	50–150 pg/mL	Reduced pace, vasoconstriction	Żebrowska et al. [20]; Žákovská et al. [21]
Mountain/High-Altitude Races	Wide range; up to 300 + pg/mL	Eccentric load, altitude, hypoxia	Hoppel et al. [31]; Le Goff et al. [23]; Bernecker et al. [22]
Experienced vs. Novice Athletes	Lower peaks in trained	Training adaptation, immune conditioning	Millet and Millet [30]; Benedetti et al. [17]
Recovery Phase (Post Race)	Returns to baseline in 24–48 h	Cytokine clearance, immune regulation	Nieman et al. [33]; Arakawa et al. [14]; Gill et al. [6]

### 3.3. C-Reactive Protein (CRP) Response

C-reactive protein (CRP), synthesized by the liver in response to IL-6 and other upstream cytokines, acts as a slower but sustained marker of systemic inflammation. While IL-6 reflects acute muscular and metabolic strain, CRP peaks later, typically 12 to 48 h post race, and often remains elevated during early recovery. CRP was measured in nearly all included studies and consistently demonstrated post-race elevation, confirming its utility as a downstream marker of systemic immune activation. CRP responses were generally moderate in shorter events (<100 km). Rubio-Arias et al. [13] reported mild elevations (2–10 mg/L) 24 h after a 50 km trail race, which returned to baseline by 48 h. Landers-Ramos et al. [19] observed a similar pattern, with transient increases in CRP among recreational runners. These responses were typically proportional to IL-6 spikes and reflected effective inflammatory resolution in well-trained individuals. CRP levels increased substantially in 100 km races and 12–24 h races. Kasprowicz et al. [9] reported values exceeding 25 mg/L by 24 h, with incomplete normalization at 48 h. Czajkowska et al. [38] and Kaufmann et al. [26] also documented elevated CRP in 100 km participants, with greater magnitude and longer persistence than in marathon comparators.

Hoppel et al. [31] observed CRP >30 mg/L in alpine runners, reflecting cumulative eccentric strain and environmental exposure. In these mid-range formats, inter-individual variability was notable, with training status and muscle resilience influencing peak and recovery values. CRP elevations were most pronounced in extreme and multiday events (>200 km). In the Spartathlon, Margeli et al. [2] recorded a 152-fold increase in CRP by race completion. Skenderi et al. [36] found that while IL-6 normalized rapidly, CRP and serum amyloid A remained elevated, highlighting the prolonged kinetics of hepatic acute-phase proteins. Shin et al. [10] reported stage-dependent increases in hs-CRP during a 622 km desert ultramarathon, with values higher after 308 and 622 km races than after 100 km. This study supports a dose–response relationship between distance and systemic inflammation. Environmental conditions also modulated CRP behavior. Gill et al. [6] observed CRP values nearly double those seen in temperate events of similar length during a 24 h desert race. Skinner et al. [12] reported persistent CRP elevation over six days of hot, arid racing. By contrast, Żebrowska et al. [20] and Žákovská et al. [21] found that cold weather events produced shorter-duration CRP responses, suggesting an environmental moderation of systemic load. Training history and race strategy influenced CRP recovery. Millet and Millet [30] found lower peaks and faster clearance in experienced runners. However, Benedetti et al. [17] and Hoppel et al. [31] showed that high CRP values could persist even in elite athletes under demanding conditions. Gajda et al. [28] reported a CRP peak of 92 mg/L after a 24 h treadmill race, among the highest recorded in controlled environments, demonstrating that metabolic duration alone can induce prolonged inflammation. In some studies, CRP served as a post-race recovery index. Benedetti et al. [32] linked elevated CRP at 48 h with delayed recovery and muscle dysfunction. Nieman et al. [40] found that NSAID use modified CRP clearance, possibly through interference with the IL-6 pathway. These findings suggest that CRP may signal incomplete recovery or excessive stress, rather than pathology per se. The CRP recovery trajectories were slower than IL-6. In most studies, IL-6 levels returned to normal within 24–48 h, while CRP remained elevated for 48–72 h or longer [5,37,40]. Despite subjective recovery, Arakawa et al. [14] found that CRP was elevated five days post-race. This dissociation underscores the importance of CRP in monitoring the late-phase systemic response. Figure 3 shows CRP kinetics across race formats, while Table 2 summarizes the key values, timing, and modulators. Figure 5 illustrates the CRP response intensity across the studies and time points, confirming its characteristic delayed elevation and persistence into the early recovery phase. CRP is a robust marker of systemic inflammatory load, making it a valuable tool for monitoring post-race stress and recovery needs. Its prolonged elevation, especially after multiday or thermally stressful events, warrants attention from clinicians and coaches overseeing training cycles. Table 3 provides an overview of CRP dynamics, highlighting its delayed but substantial increase following ultramarathon races. Peak levels typically occur 24–72 h post-race and are influenced by factors such as race intensity and environmental stress.

### 3.4. Tumor Necrosis Factor-Alpha (TNF-α) Response

Tumor necrosis factor-alpha (TNF-α) is a key pro-inflammatory cytokine involved in catabolism, immune cell activation, and systemic inflammatory cascades. However, unlike IL-6 and CRP, the TNF-α response to ultramarathon exertion was inconsistent across the studies and was often suppressed rather than elevated. This pattern suggests selective immune modulation rather than generalized activation. Drenth et al. [4] were among the first to report unchanged TNF-α levels following a six-hour ultramarathon. Nieman et al. [24] found similarly stable values after a 161 km trail race, despite significant increases in IL-6 and CRP. Jee and Jin [25] and Skottrup et al. [29] also observed no elevation of TNF-α following 100 km and coastal ultramarathon events, respectively, despite IL-6 levels peaking sharply. This dissociation supports the idea that TNF-α is subject to downregulation in endurance-trained individuals. In high-intensity multiday events, TNF-α remained suppressed or only modestly elevated.

In the Spartathlon (246 km), Skenderi et al. [36] and Margeli et al. [2] documented substantial increases in IL-6 and CRP, with no significant response in TNF-α. Shin et al. [10] reported unchanged TNF-α levels across 100 km, 308 km, and 622 km races, despite high CRP and ferritin, reinforcing that systemic TNF-α activation is not a primary feature of prolonged exertion.

Environmental stressors may influence TNF-α patterns. Gill et al. [6] found modest increases following a 24-h desert race, which coincided with elevated levels of IL-10 and sCD14, suggesting increased intestinal permeability and monocyte activation. Krzemiński et al. [35] reported a statistically significant rise after a 100 km mountain race, but the increase remained within physiological bounds. These examples imply that heat, gut stress, and oxidative load may act as cofactors, rather than direct inducers.

Training status played a limited role in TNF-α modulation. Millet and Millet [30] found no differences between novice and experienced runners during an 85 km trail race. This suggests that TNF-α suppression may be a conserved response across trained individuals, reflecting broader immune regulation rather than training-specific adaptation.

Anti-inflammatory mediators likely play a role in containing TNF-α. Nieman et al. [40] and Benedetti et al. [17] both reported concurrent elevations in IL-10 and sCD163, which are known to inhibit TNF-α synthesis. Marklund et al. [34] found similar patterns in a ski ultramarathon, with elevated levels of IL-6 and IL-10, but stable levels of TNF-α. These findings support the hypothesis that IL-6, while pro-inflammatory, may also initiate a compensatory feedback loop that selectively dampens TNF-α activity.

Ex vivo analyses further support this. Skottrup et al. [29] demonstrated that monocytes remained responsive post race, even as circulating TNF-α remained low. Jee and Jin [25] observed comparable results. This suggests that systemic suppression reflects a controlled immune strategy, rather than exhaustion or immune fatigue. TNF-α variability is best explained not by methodological inconsistency but by physiological regulation. Several studies report the co-activation of IL-10 and sCD163, suggesting that anti-inflammatory containment may selectively suppress TNF-α expression even under high exertional loads.

Controlled environment studies confirmed this trend. Gajda et al. [28] reported no increase in TNF-α after a 24-h treadmill ultramarathon, despite elevated IL-6 levels. Fallon [1], analyzing a 6-day track event, found that multiple acute-phase proteins increased while TNF-α did not. These results highlight that TNF-α is not central to the physiological stress response to ultramarathon running and may serve more as a modulated endpoint than a diagnostic signal.

Figure 4 presents typical TNF-α trajectories, and Table 3 summarizes the response range, timing, and modulators of TNF-α. Figure 5 illustrates the overall low-to-moderate TNF-α intensities across the timepoints and studies. Together, these patterns emphasize the regulatory suppression of this cytokine in trained athletes.

As shown in Table 4, TNF-α responses were generally modest and more variable compared with IL-6 and CRP. Elevations were observed in some long-distance or extreme events, but they often remained unchanged, particularly in well-trained athletes or during cold weather conditions.

### 3.5. Anti-Inflammatory Mediators and Recovery Dynamics

While IL-6 and CRP reflect the pro-inflammatory and acute phase response to ultramarathon exertion, many studies have also demonstrated a consistent activation of counter-regulatory pathways that facilitate immune recovery and inflammation resolution. These anti-inflammatory mediators, including interleukin-10 (IL-10), interleukin-1 receptor antagonist (IL-1ra), and soluble CD163 (sCD163), were measured in several studies and provide critical context for interpreting the systemic immune response. IL-10, a well-characterized regulatory cytokine, was frequently elevated following ultramarathon races. Nieman et al. [40], studying 161 km trail runners, found substantial increases in IL-10 following peaks in IL-6, suggesting a compensatory feedback loop. Marklund et al. [34] observed concurrent elevations of IL-6 and IL-10 in a 75 km ski ultramarathon, with IL-10 remaining elevated during the early recovery period. Benedetti et al. [17] reported that IL-10 rose in parallel with sCD163, a macrophage activation marker associated with the polarization of anti-inflammatory monocytes. IL-1ra followed a similar pattern. Drenth et al. [4] observed an elevation in IL-1ra without a change in TNF-α following a 60 km ultramarathon, indicating selective cytokine modulation. Benedetti et al. [17] also documented increased IL-1ra in conjunction with NT-proBNP, linking systemic inflammation with cardiac strain. Žákovská et al. [21] reported elevated IL-1ra on the second day of recovery after a cold weather ultramarathon, supporting the idea that anti-inflammatory regulation may outlast the IL-6/CRP surge. Macrophage-related markers also supported this regulatory trend. Gill et al. [6] observed increased sCD14 and sCD163 post race, along with modest elevations in IL-10 and TNF-α. Benedetti et al. [17] interpreted similar increases in sCD163 as indicative of monocyte reprogramming toward tissue repair.

These patterns align with trained immunity, where repeated exposure to physiological stress favors less inflammatory, more regulatory leukocyte responses. Several studies examined the relationship between oxidative stress and immune activation. Sadowska-Krępa et al. [41], studying runners in a 12 h event, found a concurrent elevation of IL-6 and malondialdehyde (MDA), linking cytokine signaling to lipid peroxidation. Díaz-Castro et al. [42] explored this relationship in a nutritional intervention, showing that antioxidant supplementation modulated post-race cytokine and oxidative markers without accelerating recovery. Recovery dynamics were shaped by race duration, athlete profile, and the interplay between pro- and anti-inflammatory mediators. IL-6 typically resolves within 24–48 h in most studies, including those by Nieman et al. [24], Arakawa et al. [14], and Czajkowska et al. [38].

In contrast, CRP remained elevated for 48–72 h or longer, particularly in extreme-distance or multi-stage formats [5,10,40]. Benedetti et al. [17] found that high CRP at 48 h correlated with muscle dysfunction and delayed recovery. Żebrowska et al. [20] reported sustained CRP and sCD163 elevation 3 days after a 48 h mountain event. Training status influenced, but did not guarantee, faster recovery. Millet and Millet [30] found that experienced runners returned to baseline faster than novices. However, even highly trained runners displayed prolonged inflammatory signatures in demanding conditions. Hoppel et al. [31] found persistent C-reactive protein (CRP) and N-terminal pro-B-type natriuretic peptide (NT-proBNP) in elite athletes following high-altitude races. Several studies have confirmed that recovery is not a passive decline in inflammation, but an actively regulated immunological process. Jee and Jin [25] and Skottrup et al. [29] showed that leukocytes remained responsive to ex vivo stimulation even when serum IL-6 and CRP had normalized. Goussetis et al. [43], who tracked athletes during Spartathlon recovery, found intact or enhanced function of circulating progenitor cells, reinforcing the concept of a controlled, resilient immune response. In summary, anti-inflammatory mediators such as IL-10, IL-1ra, and sCD163 play a central role in resolving systemic inflammation following ultramarathon stress. Their elevation in the absence of TNF-α supports the notion that trained athletes initiate both inflammatory and resolution cascades in parallel, allowing for efficient recovery without immune exhaustion.

## 4. Discussion

This review reveals that ultramarathon running induces a profound but well-regulated inflammatory response characterized by the sequential activation of cytokines and acute-phase proteins. To structure our synthesis, we organized biomarker responses by time course kinetics, post-race resolution patterns, and modulatory factors, including race duration, environmental stress, and training status. Our findings align with the comprehensive summary by Braschler et al. [44], who reported consistent increases in IL-6, CRP, and TNF-α across endurance events, with IL-6 showing rapid elevation, CRP peaking at 24–72 h, and TNF-α demonstrating high inter-individual variability. Rather than mimicking uncontrolled inflammatory syndromes (e.g., sepsis or trauma), this cascade reflects a dynamic physiological mechanism that initiates tissue repair, preserves immune competence, and restores homeostasis.

Among the biomarkers analyzed, IL-6 consistently emerged as the earliest and most sensitive indicator of physiological strain. IL-6 concentrations rose rapidly during competition, typically peaking near or immediately after the finish. This pattern was remarkably consistent across race distances, environmental conditions, and athlete training levels, as illustrated in Figure 2. IL-6’s dual role as both a myokine and cytokine explains its capacity to regulate both substrate mobilization and immune signaling. Higher peaks were observed in longer events (>200 km), desert races, and multiday formats, with delayed normalization in extreme efforts, such as the Spartathlon or 48-h stage races (see Table S1 and Figure 5). In line with this interpretation, Górecka et al. [45] found that the elevations in IL-6 and TNF-α during marathon running were closely associated with lipid mobilization markers, such as ANGPTL4, free fatty acids, and glycerol, reinforcing IL-6′s integrated metabolic–inflammatory function during endurance stress.

CRP followed a delayed and more sustained trajectory. Synthesized in the liver in response to IL-6, CRP concentrations typically peaked 24–48 h post race and often remained elevated for 2–3 days. This secondary phase reflects accumulated systemic stress and muscle damage rather than acute exercise strain. CRP magnitude and duration were strongly associated with race length and cumulative exposure, with the highest levels seen in multi-stage or alpine events (Figure 3). Shin et al. [10] demonstrated a stepwise increase in hs-CRP across 100 km, 308 km, and 622 km races, reinforcing the dose–response relationship between endurance load and post-race systemic inflammation. Environmental conditions also modulated CRP behavior. Gill et al. [6] observed CRP values nearly double those seen in temperate events of similar length during a 24 h desert race. Skinner et al. [44] reported persistent CRP elevation over six days of hot, arid racing. By contrast, Żebrowska et al. [20] and Žákovská et al. [21] found that cold weather events produced shorter-duration CRP responses, suggesting an environmental moderation of systemic load. Recent meta-analytic evidence by Khosravi et al. [46] and García-Hermoso et al. [47] also supports these observations, demonstrating that aerobic exercise is more effective in reducing IL-6 and CRP levels than resistance or mixed modalities, underscoring the systemic inflammatory demands of prolonged endurance formats.

Over time, regular endurance training may attenuate baseline inflammation and enhance immunoregulation. Silva and Oliveira [48] reviewed the impact of chronic aerobic training on IL-6 dynamics and immunosenescence, showing that habitual endurance activity suppresses the low-grade inflammation associated with aging. Likewise, Kazeminasab et al. [49] reported that long-term aerobic training led to significant reductions in both CRP and IL-6 levels in healthy adults, with modest effects on TNF-α. These findings support the interpretation that the observed cytokine elevations post race reflect acute, self-limiting perturbations in otherwise-adapted individuals.

## 5. Conclusions

This review confirms that ultramarathon running provokes a distinct and regulated inflammatory response, characterized by a rapid surge in IL-6 during exercise, a delayed but prolonged elevation in CRP post race, and typically modest or absent changes in TNF-α. This sequence reflects a structured immune cascade, with IL-6 initiating the response, CRP capturing accumulated stress, and TNF-α appearing restrained through compensatory mechanisms.

The consistency of this pattern across 28 studies—spanning distances, environments, and athlete profiles—underscores its physiological relevance. IL-6 typically resolves within 24–48 h, while CRP often remains elevated for 48–72 h or longer. TNF-α responses were either suppressed or negligible, suggesting that systemic containment is an adaptive feature in trained ultramarathon runners.

These findings may offer practical insights for athlete monitoring and recovery planning, although current evidence remains preliminary and subject to methodological variation. IL-6 and CRP can aid in detecting under-recovery or excessive exertional load, while TNF-α should be interpreted in the broader context of immune regulation. Ultramarathon events also serve as real-world laboratories for studying systemic inflammation under prolonged physical stress. Their ability to evoke robust but contained cytokine responses highlights the potential of these models for future immunological and performance research.

## 6. Limitations

Despite its comprehensive scope, this review is subject to several limitations. First, the included studies displayed methodological heterogeneity in terms of race formats, environmental conditions, and participant training status, which complicates direct comparisons and synthesis. Second, blood sampling schedules varied considerably, particularly in follow-up intervals, which limited the interpretation of cytokine resolution curves, especially for CRP and TNF-α.

Third, TNF-α was underreported relative to IL-6 and CRP, and when reported, it was often measured using low-sensitivity assays. Fourth, sample sizes were generally small, and female athletes, older participants, and novice runners were underrepresented. This restricts the generalizability across demographics.

Fifth, pre-race behaviors (e.g., NSAID use, sleep status, diet, and hydration) were often unreported and likely influenced cytokine responses. Sixth, although the Newcastle–Ottawa Scale was used to assess study quality, four studies were rated as moderate due to missing sampling details or the inadequate control of confounders.

Seventh, most studies did not report participant ethnicity, geographic background, or nutritional behavior in sufficient detail to support subgroup analysis. This lack of reporting limits the interpretability across populations and reflects a structural gap in the current endurance literature.

Finally, this review focused solely on IL-6, CRP, and TNF-α. Although these are the most consistently reported inflammatory markers in ultramarathon research, others, such as IL-10, IL-1ra, cortisol, and oxidative stress indices, were excluded but are worthy of further study.

## 7. Future Research Directions

This review highlights several important avenues for future research. First, longitudinal studies are needed to evaluate the effects of repeated ultramarathon participation on chronic inflammation, immune resilience, and training adaptations. The evolution of biomarker trajectories across training seasons or consecutive competitions is poorly understood.

Second, studies should include more diverse populations. Female runners, older athletes, and less-experienced individuals remain underrepresented. Understanding how sex hormones, age, and baseline fitness shape cytokine kinetics is critical.

Third, future research should broaden its scope beyond IL-6, CRP, and TNF-α. Anti-inflammatory mediators (e.g., IL-10 and IL-1ra), stress hormones (e.g., cortisol), and oxidative stress markers (e.g., MDA and GSH) may provide a more comprehensive immunological profile. Multi-omics approaches—including proteomics and metabolomics—could uncover individualized recovery signatures.

Fourth, standardized protocols for sample timing and reporting should be adopted. Including consistent post-race intervals (e.g., 0 h, 6 h, 24 h, 48 h, and 72 h) and clearly describing the race conditions and athlete behaviors would facilitate cross-study comparison and future meta-analysis.

Finally, clinical thresholds for overreaching, recovery readiness, and cardiovascular strain must be defined. IL-6 and CRP may have value not only as scientific markers but as practical tools for optimizing tapering, return-to-training, and medical oversight in endurance sports. Future research should also adopt standardized sampling protocols across athlete populations, event formats, and environmental conditions to improve the interpretability and validation of inflammatory biomarkers in endurance sports.

## Figures and Tables

**Table 2:** Summary of biomarker characteristics by race, format, and conditions.

Biomarker	Race Type	Peak Range	Time to Peak	Recovery Duration	Environmental Modulators
IL-6	<100 km	20–100 pg/mL	0–1 h	<24 h	Heat, altitude
IL-6	100–200 km	80–250 pg/mL	0–1 h	24–48 h	Terrain, metabolic load
IL-6	>200 km	500–7000 + pg/mL	0–1 h	48–72 h	Multi-stage exertion
CRP	<100 km	2–10 mg/L	24–48 h	~48 h	Mild systemic stress
CRP	100–200 km	10–40 mg/L	24–48 h	~72 h	Eccentric loading, terrain
CRP	>200 km	30–100 + mg/L	24–72 h	>72 h	Cumulative fatigue, heat
TNF-α	<100 km	5–15 pg/mL	0–6 h	Varies	Often unchanged
TNF-α	100–200 km	10–25 pg/mL	0–6 h	Varies	Gut stress, heat
TNF-α	>200 km	Often stable, 15–40 pg/mL	0–6 h	Returns to baseline	IL-10-mediated suppression

**Table 3:** Characteristics of CRP responses in ultramarathon studies.

Race Type/Condition	Typical Distance/Duration	Peak CRP Range	Key Modulators	Selected References
Single-Stage Trail Races (50–70 km)	50–70 km	2–10 mg/L	Muscle microtrauma, mild systemic stress	Rubio-Arias et al. [13]; Czajkowska et al. [38]
100 km Ultras and 12–24 h Races	100 km or 12–24 h	10–40 mg/L	Duration, terrain intensity, eccentric loading	Kasprowicz et al. [9]; Benedetti et al. [17,32]
Extreme Continuous Events (>200 km)	>200 km (e.g., Spartathlon)	30–100 + mg/L	Prolonged exposure, cumulative fatigue	Skenderi et al. [18]; Margeli et al. [2]; Shin et al. (2013) [37]
Hot Environment Races	Any distance	Often >30 mg/L	Dehydration, thermal strain, gut permeability	Gill et al. [5,6]; Skinner et al. [12]
Cold Weather Races	50–100 + km	5–20 mg/L	Reduced inflammatory drive, cooler temperatures	Żebrowska et al. [20]; Žákovská et al. [21]
Mountain/High-Altitude Races	Typically 100 + km	15–50 mg/L	Altitude, eccentric muscle damage, hypoxia	Hoppel et al. [31]; Le Goff et al. [23]; Bernecker et al. [22]
Experienced vs. Novice Athletes	All formats	Lower CRP in trained	Enhanced recovery, reduced tissue damage	Millet and Millet [30]; Benedetti et al. [17]
Recovery Phase (Post Race)	24–72 h post race	Peaks at 24–48 h	Hepatic delay, IL-6-driven acute phase response	Arakawa et al. [14]; Nieman et al. [24]; Gill et al. [4,5]

**Table 4:** Characteristics of TNF-α responses in ultramarathon studies.

Race Type/Condition	Typical Distance/Duration	Peak TNF-α Range	Key Modulators	Selected References
Single-Stage Trail Races (50–70 km)	50–70 km	5–15 pg/mL	Exercise duration, sampling timing, baseline fitness	Czajkowska et al. [38]; Rubio-Arias et al. [13]
100 km Ultras and 12–24 h Races	100 km or 12–24 h	10–25 pg/mL	Cumulative load, oxidative stress	Kasprowicz et al. [9]; Benedetti et al. [17,32]
Extreme Continuous Events (>200 km)	>200 km (e.g., Spartathlon)	Often stable, 15–40 pg/mL	Immunological regulation, physiological exhaustion	Skenderi et al. [18]; Margeli et al. [2]; Shin et al. [10]
Hot Environment Races	Any distance	10–30 pg/mL (variable)	Gut permeability, heat stress, monocyte activation	Gill et al. [6]; Skinner et al. [12]
Cold Weather Races	50–100 + km	5–15 pg/mL	Reduced systemic activation, thermal moderation	Żebrowska et al. [20]; Žákovská et al. [21]
Mountain/High-Altitude Races	Typically 100 + km	Typically <30 pg/mL	Hypoxia, eccentric strain, immune containment	Hoppel et al. [31]; Le Goff et al. [23]; Bernecker et al. [22]
Experienced vs. Novice Athletes	All formats	Lower in trained athletes	Conditioning, immunoregulation	Millet and Millet [30]; Benedetti et al. [17]
Recovery Phase (Post Race)	0–24 h post race	Often returns to baseline	TNF-α suppression via IL-10 and IL-1ra	Jee and Jin [25]; Skottrup et al. [29]; Drenth et al. [4]

## Data Availability

Data are available from the authors.

## Supplementary Materials

The following supporting information can be downloaded at: https://www.mdpi.com/article/10.3390/ijms26136317/s1. References [1,2,4,5,7,9,12,13,14,17,19,21,22,23,25,28,30,31,32,34,35,37,38,40,41,42,43] are cited in Supplementary Materials.
