# Supplementary material for: Inflammatory Response to Ultramarathon Running: A Review of IL-6, CRP, and TNF-α
Source: Int J Mol Sci. 2025 Jun 30;26(13):6317. doi: 10.3390/ijms26136317 (PMC12250383; doi:10.3390/ijms26136317)
Supplement: Supplementary file 1 [file ijms-26-06317-s001.zip › ijms-3627321-supplementary.pdf]

Table S1. Newcastle–Ottawa Scale (NOS) Quality Assessment of the 28 Studies Included in the Systematic Review

Study	Selection (0–4)	Comparability (0–2)	Outcome (0–3)	Total Score (0–9)	Quality Rating
Arakawa et al. (2016)[14]	3	2	3	8	High
Benedetti et al. (2018)[32]	4	2	2	8	High
Benedetti et al. (2021)[17]	3	1	2	6	Moderate
Bernecker et al. (2013)[22]	3	1	2	6	Moderate
Chlíbková et al. (2017)[21]	3	2	2	7	High
Costa et al. (2014)[7]	4	2	2	8	High
Czajkowska et al. (2020)[38]	3	2	2	7	High
Drenth et al. (1995)[4]	3	1	2	6	Moderate
Díaz-Castro et al. (2012)[42]	3	2	2	7	High
Fallon (2001)[1]	4	1	3	8	High
Gajda et al. (2020)[28]	3	1	3	7	High
Gill et al. (2015)[5]	3	1	2	6	Moderate
Goussetis et al. (2009)[43]	3	1	3	7	High
Hoppel et al. (2019)[31]	3	1	3	7	High
Jee et al. (2012)[25]	4	2	3	9	High
Kasprowicz et al. (2013)[9]	3	2	3	8	High
Krzemiński et al. (2016)[35]	4	2	2	8	High
Landers-Ramos et al. (2022)[19]	4	2	3	9	High
Le Goff et al. (2020)[23]	4	2	2	8	High
Margeli et al. (2005)[2]	3	1	3	7	High
Marklund et al. (2013)[34]	4	2	3	9	High
Millet & Millet (2012)[30]	3	2	3	8	High
Nieman et al. (2005)[40]	4	1	2	7	High
Rubio-Arias et al. (2018)[13]	4	2	3	9	High
Sadowska-Krępa et al. (2021)[41]	4	1	2	7	High
Shin et al. (2023)[37]	4	2	3	9	High
Skinner et al. (2021)[12]	4	1	2	7	High
Žákovská et al. (2017)[21]	4	2	3	9	High
